# Accuracy Improvement of Attitude Determination Systems Using EKF-Based Error Prediction Filter and PI Controller

**DOI:** 10.3390/s20144055

**Published:** 2020-07-21

**Authors:** Farzan Farhangian, Rene Landry

**Affiliations:** LASSENA Laboratory, Department of Electrical Engineering, Ecole de Technologie Superieure, Montreal, QC H3C 1K3, Canada; renejr.landry@etsmtl.ca

**Keywords:** attitude and heading reference system, extended Kalman filter, inertial navigation, MEMS, inertial measurement unit, error prediction

## Abstract

Accurate attitude and heading reference system (AHRS) play an essential role in navigation applications and human body tracking systems. Using low-cost microelectromechanical system (MEMS) inertial sensors and having accurate orientation estimation, simultaneously, needs optimum orientation methods and algorithms. The error of attitude estimation may lead to imprecise navigation and motion capture results. This paper proposed a novel intermittent calibration technique for MEMS-based AHRS using error prediction and compensation filter. The method, inspired from the recognition of gyroscope’s error and by a proportional integral (PI) controller, can be regulated to increase the accuracy of the prediction. The experimentation of this study for the AHRS algorithm, aided by the proposed prediction filter, was tested with real low-cost MEMS sensors consists of accelerometer, gyroscope, and magnetometer. Eventually, the error compensation was performed by post-processing the measurements of static and dynamic tests. The experimental results present about 35% accuracy improvement in attitude estimation and demonstrate the explicit performance of proposed method.

## 1. Introduction

In the last decade, the development of the strap-down inertial navigation systems (INS) in various applications, such as unmanned aerial vehicles (UAV), missile guidance, body tracking systems, and underwater automatic vehicles, has led to the essential need to design low-weight, low-cost, and low-power-consumption INS with reliable and robust accuracy. With advancements in microelectromechanical system (MEMS) technologies, using the MEMS-based inertial measurement units (IMU) has become more prevalent due to their low-cost, low power consumption, and small size [1]. The main objective of AHRS is to estimate how the orientation of body frame relates to the navigation frame, which can be shown by quaternion vector or Euler angles. For researchers, the difficulties of the system are related to obtaining the nondivergent output and avoiding the error accumulation, which may come due to the time-integration of gyroscope outputs [1,2].

One of the most substantial problems in AHRS using the MEMS-based IMU is their dependency on drift and measurement errors, which lead to divergence of attitude and heading error over long time experiments. This paper presented a new method for predicting and bounding the AHRS error. The method attempted to anticipate the behavior of the error using mathematical investigation on the gyroscope’s drift. Consequently, the error of each channel, namely roll, pitch, and yaw were estimated in low-amplitude signals. Also, a regulator was considered that consisted of a constant shifter and a proportional integral (PI) controller for each element of the orientation. The best error estimation was obtained by tuning the parameters of the filter, and eventually, the error was subtracted from the output of EKF-based AHRS. The main idea of the method is from nature of the orientation error in INS sensors, which only need the measurement of gyroscope. To evaluate the proposed method, a low-cost, nine-degree-of-freedom (DoF) MEMS-based IMU was used, consisting of a three-axis gyroscope, three-axis accelerometer, and three-axis magnetometer. A Simulink design of the method and the EKF model was used in laboratory experiments with the calibrated data from the mentioned IMU module. The results demonstrate the improvement in precision of AHRS in all the channels of roll, pitch, and yaw with regard to proper adjustment of the filter’s parameters.

In Section 2, we present an overview of prior works in the literature, relevant to our research, and the background of a time-variant covariance quaternion-based EKF and its parameters is considered. In Section 3, we describe an attitude error prediction and compensation filter as an accuracy improvement method and elaborate on the PI controller and regulator. The experiments and the results are presented in Section 4 and the conclusions from the paper and future directions are presented in Section 5.

## 2. Related Works

Research studies on designing an accurate AHRS with minimum error could be categorized in three main topics, namely complementary filters (CF), optimization approaches, and Kalman filter (KF)-based solutions. The authors of [3,4] have considered AHRS improvement using CF and MEMS sensors. A more completed AHS was designed and implemented by the authors of [5] based on explicit CF, which showed great capability for implementation in embedded hardware [5]. In these papers, although the systems showed acceptable real-time performance, the drift estimation in passive and direct CFs were not robust enough in highly dynamic experiments. Apart from that, the system demonstrated dependency to methods of initialization. Adaptive Kalman filter (AKF) [6], extended Kalman filter (EKF) [7], and dual Kalman filter (DKF) methods [8] have been performed for orientation estimation with a basic model of the system. A precise stable Kalman-based AHRS was designed as an approach of the simultaneous localization and mapping (SLAM) applications by the authors of [9]. Furthermore, an EKF using a depth measurement for underwater AHRS and unscented Kalman filter (UKF) methods was investigated by the authors of [10,11]. As the attitude estimators require the precise model and noise covariance determiner, a novel time-varying noise covariance EKF was designed and experimented. The designed filter considered changing the noise covariance matrix of the system’s model to identify the magnitude of the angle between the estimated gravitational and measured acceleration [12]. In addition, determination of this angle and the orientation between the body and the navigational coordinates can be considered as an optimization problem. The quaternion-based optimization approach was studied by the authors of [13] using the gradient descent algorithm, known as Madgwick, with magnetic angular rate and gravity (MARG) sensors [14]. The mentioned works have had great improvements in attitude and heading estimation using kinds of Kalman-based systems. However, they did not analyze the behavior of error in noisy measurements, which is prevalent in low-cost AHRS designs. As a result, in highly dynamic maneuvers, fast angular rates, and noisy environments, the outputs can be diverged through time.

Low-cost MEMS-based sensors, especially the magnetometers, are required to be calibrated deterministically and stochastically. A KF-based AHRS method was studied by the authors of [15] in environments with high magnetic distortion. Also, some efforts have been performed to use attitude determination methods in healthcare applications, such as the fall detection of elderly persons [16], motion capture for sport activity analysis [17], and shoulder injury prevention [18]. The mentioned works have only considered special scenarios, which means they did not show the potential of their works to be expanded in all medical situations and sport activities. Wearable low-cost and lightweight IMUs were used in those applications. A comprehensive comparison between CF, UKF, EKF, Madgwick, and Mahony by the authors of [19] showed that the minimum AHRS error between CF and Kalman-based algorithms is accounted for by the EKF, with better results in attitude and position estimations. The authors of [20] compared Mahony and Madgwick and demonstrated that there is no significant difference between them. Apart from the mentioned filter-based AHRS algorithms, some researches have investigated the use of integration of AHRS with GPS signals to estimate more precise Euler angles. The method has shown significant results in airspace applications, but the method is significantly affected by pseudorange measurements of GPS and cannot respond to GPS-challengeable situations [21,22,23].

### 2.1. EKF Model and Parameters

There are different orientation representations between the body and navigation frame. This paper considered the quaternion-based equations because of their nonsingularity and lower computation complexity compared to directed cosine matrix (DCM) with nine integration equations [12,24]. The system model of EKF, defined with state vector $x_{k}$, is formulated in Equation (1).

(1) $$x_{k}=F_{k}x_{k-1},\;\;\;x_{k}={\left[\begin{array}{cc} q_{1} & q_{2} \end{array}\;\;\;\;\begin{array}{cc} q_{3} & q_{4} \end{array}\right]}^{T},$$

where $q_{1}$, $q_{2}$, $q_{3}$, and $q_{4}$ are the elements of quaternion vector of $q=q_{1}+q_{2}\vec{i}+q_{3}\vec{j}+q_{4}\vec{k}$, and model matrix of $F_{k}$ is defined as Equations (2) and (3). $\bar{\bar{\omega }}$ is the rotation angle for a time step, and matrix μ is the skew symmetric of quaternion form of angular velocity vector [12,25].

(2) $$F_{k}=\cos\left(\frac{\bar{\bar{\omega }}}{2}\right)I+\frac{\sin\left(\frac{\bar{\bar{\omega }}}{2}\right)}{\frac{\bar{\bar{\omega }}}{2}}\mu \;dt,\mu =\left[\begin{array}{c} 0\\ \begin{array}{c} \omega_{x}\\ \begin{array}{c} \omega_{y}\\ \omega_{z} \end{array} \end{array} \end{array}\;\;\;\;\begin{array}{c} -\omega_{x}\\ \begin{array}{c} 0\\ \begin{array}{c} -\omega_{z}\\ \omega_{y} \end{array} \end{array} \end{array}\;\;\;\;\begin{array}{c} -\omega_{y}\\ \begin{array}{c} \omega_{z}\\ \begin{array}{c} 0\\ -\omega_{x} \end{array} \end{array} \end{array}\;\;\;\;\begin{array}{c} -\omega_{z}\\ \begin{array}{c} -\omega_{y}\\ \begin{array}{c} \omega_{x}\\ 0 \end{array} \end{array} \end{array}\right],$$

(3) $$\bar{\bar{\omega }}=norm\;\left({\left[\begin{array}{cc} \omega_{x} & \omega_{y} \end{array}\;\;\;\;\omega_{z}\right]}^{T}\right)\cdot dt=\sqrt{{\left(\omega_{x}\cdot dt\right)}^{2}+{\left(\omega_{y}\cdot dt\right)}^{2}+{\left(\omega_{z}\cdot dt\right)}^{2}},$$

For the measurement model, the magnetometer and acceleration data are considered as the EKF measurement, as defined in Equation (4). $H_{k}$ is the measurement matrix, and $z_{k}$ consists of two elements, namely the normalized specific force and normalized horizontal component of the geomagnetic field. The first one is achieved from acclerometer and the second one from magnetometer output [12,25].

(4) $$z_{k}=h\left(x_{k}\right),\;\;\;\;z_{k}={\left[\begin{array}{cc} {\bar{\bar{s}}}_{b} & {\bar{\bar{m}}}_{b} \end{array}\right]}^{T}\;,$$

where ${\bar{\bar{s}}}_{b}$ and ${\bar{\bar{m}}}_{b}$ are considered as ${\bar{\bar{s}}}_{b}=\frac{s_{b}}{\left\|s_{b}\right\|}$ and ${\bar{\bar{m}}}_{b}=a\times b$ [12]. The b and n subscripts show the body and navigation coordinates. The ${\bar{\bar{m}}}_{b}$ is calculated from cross-product of vectors a and b. This calculation is necessary because the geomagnetic field is not parallel to the earth. Fortunately, the obtained ${\bar{\bar{m}}}_{b}$ is parallel to the magnetic north [12]. Parameters a and b are defined in Equations (5) and (6) [12].

(5) $$a=\frac{g_{b}}{\left\|g_{b}\right\|}=C_{n}^{b}\frac{g_{n}}{\left\|g_{n}\right\|},$$

(6) $$b=\frac{g_{b}\times m_{b}\;}{\left\|g_{b}\times m_{b}\right\|},$$

As mentioned before, the measurement model predicts the gravitational acceleration and geomagnetism in the body frame. The predicted measurement is included two elements of ${\bar{\bar{g}}}_{b}$ and ${\bar{\bar{m}}}_{b}$ which are obtained from Equation (7). The horizontal element of ${\bar{\bar{m}}}_{b}$ is considered as the magnetometer measurement [12].

(7) $${\hat{z}}_{k}={\left[\begin{array}{cc} {\bar{\bar{g}}}_{b} & {\bar{\bar{m}}}_{b} \end{array}\right]}^{T},$$

$${\hat{z}}_{k}={\left[\begin{array}{cc} C_{n}^{b}{\left[\begin{array}{ccc} 0 & 0 & -1 \end{array}\right]}^{T} & C_{n}^{b}{\left[\begin{array}{ccc} 1 & 0 & 0 \end{array}\right]}^{T} \end{array}\right]}^{T},$$

The conversion matrix $C_{n}^{b}$ between navigation to body frame is obtained from predicted quaternion states with following the Equation (8). The simplified form of measurement matrix is considered in Equation (9) [12,25].

(8) $$C_{b}^{n}=\left[\begin{array}{ccc} q_{1}^{2}+q_{2}^{2}-q_{3}^{2}-q_{4}^{2} & 2\left(q_{2}q_{3}+q_{1}q_{4}\right) & 2\left(q_{2}q_{4}-q_{1}q_{3}\right)\\ 2\left(q_{2}q_{3}-q_{1}q_{4}\right) & q_{1}^{2}-q_{2}^{2}+q_{3}^{2}-q_{4}^{2} & 2\left(q_{3}q_{4}+q_{1}q_{2}\right)\\ 2\left(q_{2}q_{4}+q_{1}q_{3}\right) & 2\left(q_{3}q_{4}+q_{1}q_{2}\right) & q_{1}^{2}-q_{2}^{2}-q_{3}^{2}+q_{4}^{2} \end{array}\right],$$

(9) $$z_{k}=H_{k}x_{k}=\left[\begin{array}{c} q_{3}\\ \begin{array}{c} -q_{2}\\ \begin{array}{c} -q_{1}\\ \begin{array}{c} q_{1}\\ \begin{array}{c} -q_{4}\\ q_{3} \end{array} \end{array} \end{array} \end{array} \end{array}\;\;\;\;\begin{array}{c} -q_{4}\\ \begin{array}{c} -q_{1}\\ \begin{array}{c} q_{2}\\ \begin{array}{c} q_{2}\\ \begin{array}{c} q_{3}\\ q_{4} \end{array} \end{array} \end{array} \end{array} \end{array}\;\;\;\;\begin{array}{c} q_{1}\\ \begin{array}{c} -q_{4}\\ \begin{array}{c} +q_{3}\\ \begin{array}{c} -q_{3}\\ \begin{array}{c} q_{2}\\ q_{1} \end{array} \end{array} \end{array} \end{array} \end{array}\;\;\;\;\begin{array}{c} -q_{2}\\ \begin{array}{c} -q_{3}\\ \begin{array}{c} -q_{4}\\ \begin{array}{c} -q_{4}\\ \begin{array}{c} -q_{1}\\ q_{2} \end{array} \end{array} \end{array} \end{array} \end{array}\right]x_{k}\;,$$

Determination of the EKF parameters is required before the designing process. The important parameters of EKF are the measurements of noise covariance $R_{k}$, process noise covariance $Q_{k}$, and the initialization parameters comprised of initial states vector $x_{0}$ and initial error covariance $P_{0}$. Initialization considers the initial orientation of the system, which can be characterized as wahba’s problem. The most effective approach to the problem is the triad algorithm [26,27,28,29,30]. The algorithm deliniates the angle between two nonparallel vectors. To initialize the attitude determination system, the gravitional acceleration of the two vectors and the magnetic field of the earth are defined as, and m, respectively. The vectors are obtained from transforming the magnetometer and accelerometer’s output from the body frame to the navigation frame with multiplying each one with $C_{b}^{n}$ [26,27,28,29].

Two orthogonal matrices, from the body frame and from the navigation frame, are determined to calculate the transformation matrix of $C_{n}^{b}$ for attitude initialization. The orthogonal matrices are defined as $M_{b}$ and $M_{n}$, which are calculated as Equations (10) and (11) [12].

(10) $$M_{b}=\left[\begin{array}{ccc} \frac{s_{b}}{\left\|s_{b}\right\|} & \frac{s_{b}\times m_{b}\;}{\left\|s_{b}\times m_{b}\right\|} & \left(\frac{s_{b}}{\left\|s_{b}\right\|}\right)\times \left(\frac{s_{b}\times m_{b}\;}{\left\|s_{b}\times m_{b}\right\|}\right) \end{array}\right],$$

(11) $$M_{n}=\left[\begin{array}{ccc} \frac{g_{n}}{\left\|g_{n}\right\|} & \frac{g_{n}\times m_{n}\;}{\left\|g_{n}\times m_{n}\right\|} & \left(\frac{g_{n}}{\left\|g_{n}\right\|}\right)\times \left(\frac{g_{n}\times m_{n}\;}{\left\|g_{n}\times m_{n}\right\|}\right) \end{array}\right],$$

According to the fact that $M_{n}=C_{b}^{n}M_{b}$ and $C_{b}^{n}=M_{n}M_{b}^{T}$, the $C_{b}^{n}$ is calculated as the matrix in Equation (12).

(12) $$C_{b}^{n}=\left[\begin{array}{ccc} C_{11} & C_{12} & C_{13}\\ C_{21} & C_{22} & C_{23}\\ C_{31} & C_{32} & C_{33} \end{array}\right],$$

Eventually, the parameters of quaternion vactor for the attitude initialization, q(0), are obtained in Equations (13) and (14), as explained completely by the authors of [31].

(13) $$q\left(0\right)={\left[q_{1}\;\;\;\;\frac{1}{4q_{1}}\left(C_{32}-C_{23}\right)\;\;\;\;\frac{1}{4q_{1}}\left(C_{13}-C_{31}\right)\;\;\;\;\frac{1}{4q_{1}}\left(C_{21}-C_{12}\right)\right]}^{T},$$

(14) $$q_{1}=0.5\sqrt{1+C_{11}+C_{22}+C_{33}}\;,$$

After the initialization of the states vector $x_{0}$ and the error covariance matrix $P_{0}$, EKF needs to update the estimated states and estimated covariances. The measurement noise covariance was selected to vary with time with regard to *α*, which is the magnitude of the angle between the estimated gravitional acceleration, $g_{n}$, and the measured specific force, $s_{b}$ [12]. The measurement noise covariance matrix, $R_{k}$, in Equation (15), consists of the measured noise covariances of the acceleration and magnetometer sensors [12].

(15) $$R_{k}=\left[\begin{array}{cc} UI_{3\times 3} & 0_{3\times 3}\\ 0_{3\times 3} & cUI_{3\times 3} \end{array}\right],$$

where U changes as a results of the variation in *α*. The weight value, $K_{r}$, and the constant shifter, $K_{c}$, satisfy the performance accuracy of the attitude estimation algorithm [12].

(16) $$U=K_{c}+K_{r}\alpha ,\;\;\;\;\;\;\;\alpha =\cos^{-1}(\frac{\left(C_{n}^{b}g_{n}\right)\cdot s_{b}\;}{\left\|g_{n}\right\|\cdot \|s_{b}\|}),$$

(17) $$Q=\frac{1}{4}\;\vartheta \vartheta^{T}\;,\;\;\vartheta =\left[\;\begin{array}{c} -q_{2}\\ \begin{array}{c} q_{1}\\ \begin{array}{c} q_{4}\\ -q_{3} \end{array} \end{array} \end{array}\;\;\;\;\begin{array}{c} -q_{3}\\ \begin{array}{c} -q_{4}\\ \begin{array}{c} q_{1}\\ q_{2} \end{array} \end{array} \end{array}\;\;\;\;\begin{array}{c} -q_{4}\\ \begin{array}{c} q_{3}\\ \begin{array}{c} -q_{2}\\ q_{1} \end{array} \end{array} \end{array}\;\right],$$

The matrix Q is defined in Equation (17), assuming that the gyroscope uses the white gaussian noise model [25]. The proposed EKF with the time-varying noise covariance was considered for the attitude estimation in the AHRS. In the following section, the main innovation of this study regarding to prediction and compensation filter is described in detail.

## 3. Materials and Methods

The method considered in this study was based on the error prediction filter, which uses the output of AHRS algorithm inspired by proposed EKF-based method proposed by the authors of [12]. The mentioned AHRS algorithm was chosen due to its adaptability, precision, and time-varying noise covariance. The main objective of the proposed method was to improve the accuracy of the AHRS algorithms. Also, the designed filter can work with all kinds of orientation estimation methods. The attitude error prediction filter are described in Section 3.1, and the PI controller and regulator are described in Section 3.2.

**Figure 1:**
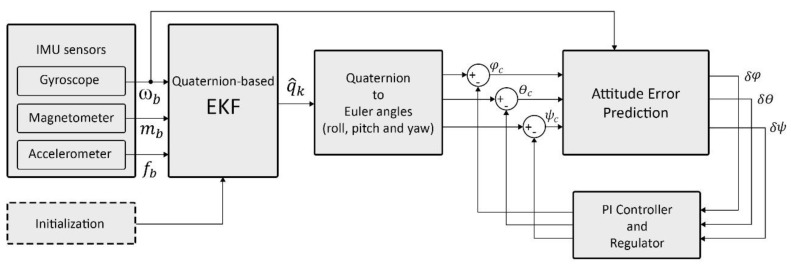
Overview of the attitude and heading reference system (AHRS) method with error prediction filter, proprtional integral (PI) controller, and regulator.

The complete idea of paper is demonstrated in Figure 1. A 9-DoF MEMS-based IMU, consisting of accelerometers, gyroscopes, and magnetometers, was connected to the quaternion-based EKF system. The system used the gyroscopes data in the error prediction filter. Also, the accelerometer and magnetometer data were defined as EKF measurements. The quaternion-based EKF, as the state vector of the system, considered the four-dimensional quaternion vector. After the estimation of the state vector by EKF, the quaternions were transformed to Euler angles of roll, pitch, and yaw. As the initialization of the EKF was required, the initialization process is described in Section 2.1. As presented in Figure 1, the prediction error filter needs the gyroscope’s angular rates to estimate and predict the orientation error. In the following steps, PI controller and regulator tune the filter output to obtain more accurate compensation.

### 3.1. Attitude Error Prediction Filter

The attitude error model is characterized as Equation (18), comprised of the roll error (*δφ*), pitch error (*δθ*), and yaw error (*δψ*). The model depends on the conversion matrix between the body and the navigation frames, error of the angular velocity in the body frame, and the proportions of attitude in time step *k*.

(18) $${\dot{\Psi }}^{N}=-C_{B}^{N}\delta \omega_{IB}^{B}-\omega_{IN}^{N}\times \Psi^{N},$$

where $\Psi^{N}={\left[\begin{array}{ccc} \delta \varphi & \delta ϴ & \delta \psi \end{array}\right]}^{T}$ and $\delta \omega_{IB}^{B}$ are the vector of error for the angular rate measurement from gyroscope, and $\omega_{IN}^{N}\times$ is the skew symmetric form of the angular velocity vector in the navigation frame.

(19) $$\omega_{IN}^{N}=C_{B}^{N}\omega_{IB}^{B}={\left[\begin{array}{ccc} \omega_{IN}^{N}\left(x\right) & \omega_{IN}^{N}\left(y\right) & \omega_{IN}^{N}\left(z\right) \end{array}\right]}^{T},$$

(20) $$\omega_{IN}^{N}\times =\left[\begin{array}{ccc} 0 & -\omega_{IN}^{N}\left(z\right) & \omega_{IN}^{N}\left(y\right)\\ \omega_{IN}^{N}\left(z\right) & 0 & -\omega_{IN}^{N}\left(x\right)\\ -\omega_{IN}^{N}\left(y\right) & \omega_{IN}^{N}\left(x\right) & 0 \end{array}\right],$$

For the simplification of the equations, we assumed that the error of the angular velocity from the gyroscope was near to zero. Equation (18) can be reduced to Equation (21), as shown below:

(21) $${\dot{\Psi }}^{N}=-\omega_{IN}^{N}\times \Psi^{N},$$

To solve this integration equation, the trapezoidal method was considered. The right side of the equation, as a time-varying function $f\left(t,\Psi^{N}\right)$, can be rewritten as the following Equation [24]:

(22) $$\frac{d\Psi^{N}}{dt}=f\left(t,\Psi^{N}\right),$$

(23) $$\Psi^{N}\left(t\right)=\int_{0}^{t}f\left(t,\Psi^{N}\right)dt,$$

(24) $$\Psi^{N}\left(t+k\right)=\int_{0}^{t}f\left(\tau ,\Psi^{N}\right)dt+\int_{t}^{t+k}f\left(\tau ,\Psi^{N}\right)dt,$$

**Figure 2:**
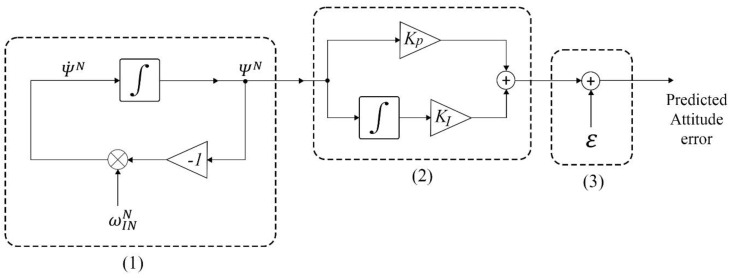
Block diagram of attitude error prediction, PI controller, and regulator. (**1**) Attitude error prediction; (**2**) PI controller; (**3**) regulator.

By approximating the Equation (24), the final discretized formula of $\Psi^{N}$ in the time step *k* can be calculated by Equation (25). The block diagram of the attitude error prediction filter is shown in Figure 2.

(25) $$\Psi_{k}^{N}=\Psi_{k-1}^{N}+\frac{h}{2}\;\left({\dot{\Psi }}_{k}^{N}+{\dot{\Psi }}_{k-1}^{N}\right),$$

Herein, the filter needs to be initialized with the amounts of $\Psi_{0}^{N}$ and ${\dot{\Psi }}_{0}^{N}$. Also, h is the system sampling time step. The initial amount of attitude error can be selected from the drift bias value in the specification of the gyroscope, and the initial change rate of attitude error can be a zero vector. The obtained $\Psi^{N}$ can be countinuous again as the error prediction signal.

### 3.2. PI Controller and Regulator

The calculated error signal $\Psi^{N}\left(t\right)$ is the prediction of the attitude error in three channels, namely roll, pitch and yaw. The proper PI controller can support the prediction while responding to the high-frequency changes in the error. Also, it can adjust the prediction to the estimated attitude from EKF system. The regulator, as a constant shifter, can regulate the final signal aligned to the estimated attitude. The standard form of PI controller was defined as Equation (26).

(26) $$u\left(t\right)=K_{P}e\left(t\right)+K_{I}\int_{0}^{t}e\left(t\right)dt,$$

Therefore, the estimated $\Psi^{N}$ from the error prediction filter is the PI controller input. The final anticipated error signal after using the regulator was calculated by:

(27) $$u\left(t\right)=\left[\begin{array}{c} K_{P\_1}\\ K_{P\_2}\\ K_{P\_3} \end{array}\right]\circ \Psi^{N}+\left[\begin{array}{c} K_{I\_1}\\ K_{I\_2}\\ K_{I\_3} \end{array}\right]\circ \int_{0}^{t}\Psi^{N}dt+\left[\begin{array}{c} \varepsilon_{1}\\ \varepsilon_{2}\\ \varepsilon_{3} \end{array}\right],$$

where the (∘) is Hadmard (element-wise) product of vectors; parameters of $K_{P\_1}$, $K_{P\_2}$, and $K_{P\_3}$ are the proportion gains of PI controller; parameters of $K_{I\_1}$, $K_{I\_2}$, and $K_{I\_3}$ are the derivative gain of each channel; and $\varepsilon_{1}$, $\varepsilon_{2}$, and $\varepsilon_{3}$ are three constant values for the regulation of each roll, pitch, and yaw channel. If the final regulated signal is considered as two parts (the PI part and the regulator part), the signal can be rewritten as:

(28) $$u\left(t\right)=p\left(t\right)+{\left[\begin{array}{ccc} \varepsilon_{1} & \varepsilon_{2} & \varepsilon_{3} \end{array}\right]}^{T},\;\;\;\;\;\;\;{\left[\begin{array}{ccc} \varepsilon_{1} & \varepsilon_{2} & \varepsilon_{3} \end{array}\right]}^{T}=-mean\;value\;\left({\left[\begin{array}{ccc} p_{1}\left(t\right) & p_{2}\left(t\right) & p_{3}\left(t\right) \end{array}\right]}^{T}\right),\;\;$$

The elements of the regulator vector were obtained from each bias signal according to the fact that their mean value should be zero over the experimental duration. The regulator constants were measured for the duration of the recorded data in the post-processing experiments.

The PI controller removes the steady state error, resulting in a standalone proportional controller. However, it may affect the speed of the response. For correcting the outputs of divergent systems using the PI controller, some criterias can be considered, namely rise time, overshooting, and transient response. Some investigation on overal stability of the system may also be needed. Therefore, determining the constant gains of PI controller is dependent on the precision of the sensors, drift of the gyroscopes, and overal stability of the system during the experiment.

## 4. Experimental Results and Discussion

### 4.1. Static Tests and Results 

**Figure 3:**
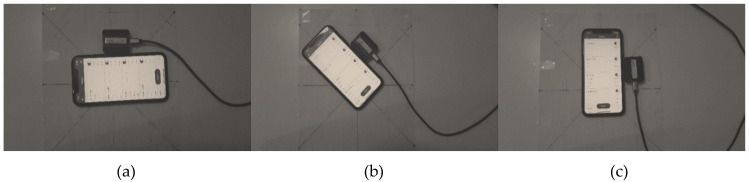
Module MPU-9250 and smartphone iPhone 11 rotation trajectory for heading test: (**a**) 180 heading degree; (**b**) 135 heading degree; (**c**) 90 heading degree.

Three experiments were performed to validate the proposed method in static mode, and today’s orientation determination technology of smartphones were considered as true attitude references. We selected a smartphone as an orientation reference because of their accessibility, MATLAB and Simulink compatibility, and precise orientation estimation in static mode [32]. The iPhone 11 and Samsung S10 were chosen for comparison. These tests were performed on a table in which the main degrees and the true north were marked as −135, −90, −45, 0, 45, 90, 135, and 180 (see Figure 3). The zero degree for the heading test was fixed to the true navigation north. To compare the selected smartphones, three special trajectories were followed by locating both smartphones at the same time in each marked angle to obtain their angle error in static mode. Each trajectory was designed for one of the channels, and the complete experimental test was performed in three different scenarios for each angle. The results of root mean square error (RMSE) for the attitude determiner of each smartphone are shown in Table 1.

Because of minimum RMSE for iPhone, this smartphone was chosen as the orientation reference of our experiments. All tests were done using data of IMU from MPU-9250, which, in this study, is referred to as “module.” The proposed EKF-based AHRS algorithm was implemented in MATLAB and estimated the final orientation of the module from the recorded data in various durations between 60 s to 100 s. The module was connected to the laptop with a USB cable, and the data of magnetometer, gyroscope, and accelerometer were recorded by the MATLAB-based serial reader. Also, data of iPhone’s IMU was recorded in the MATLAB cloud with the “MATLAB mobile” iOS application. The smartphone and module were located on the degree marked table at the same time, one time along the z axis, one time along the y axis, and finally, along the x axis. To obtain more accurate results, the trajectory for each experiment were selected in a way that allowed all angles to be measured. Figure 3 demonstrates marked table for the heading experiment, with the module and the iPhone rotated in the same trajectory on the mentioned marked table.

**Figure 4:**
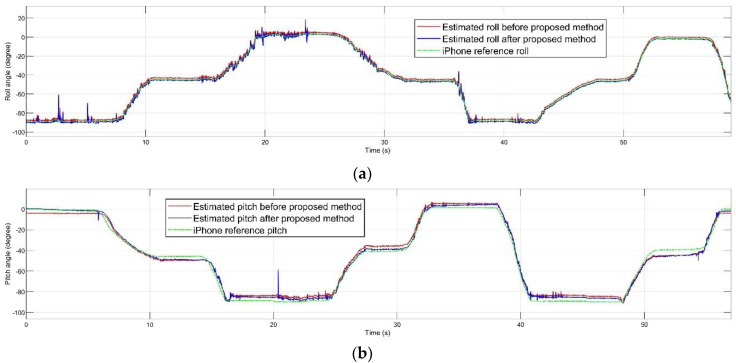
Estimated attitude before and after proposed method compared to the iPhone reference: (**a**) roll; (**b**) pitch.

**Figure 5:**
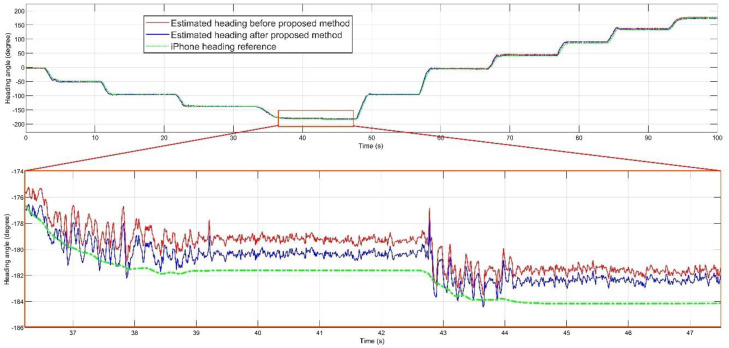
Estimated heading, before and after proposed method compared to the iPhone reference.

Figure 4 and Figure 5 shows the estimated roll, pitch, and heading angles, respectively. The estimated orientation from the time-varying noise covariance EKF-based AHRS method before and after performing the error prediction filter, PI controller, and regulator are shown. It can clearly be seen that the overall estimation was improved in many periods of time, making the total estimation more accurate. For each experiment, gains of the PI controller and amounts of the regulator are mentioned in the Table 2.

**Figure 6:**
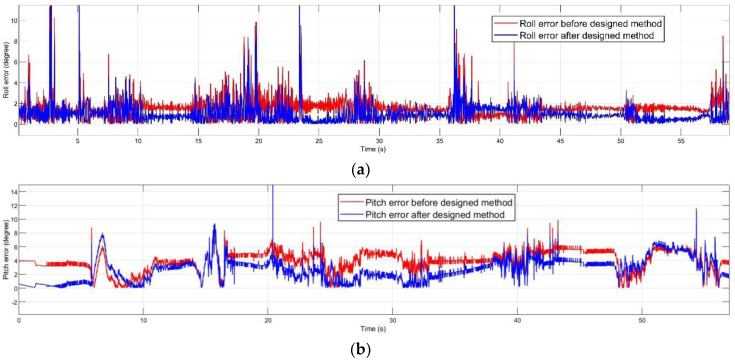

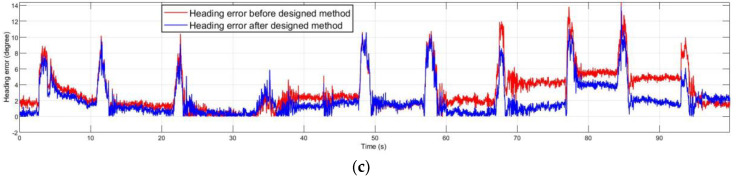
Absolute error before and after performing the proposed filter: (**a**) roll; (**b**) pitch; (**c**) heading.

Parameters of the PI controller and the constants of regulator vector were completely different in every experiment. Each variable had its own scale, as can be interpreted from Table 2. Apart from regulation constant values, which are predictable with mean value of each signal, PI parameters should be selected separately in each experiment. The highest amount of the integral gain was accounted for the pitch with the maximum error, as described in Table 3. For better understanding of the impact of the proposed compensation filter, Figure 6 demonstrates the absolute error between each estimated angle and iPhone reference orientation before and after performing the filter.

The proposed method utilized the gyroscope error model and the trapezoidal method to solve the integral equations of the filter, and finally, to obtain the signal of error for each attitude channel. The error signal was adjusted with the PI controller. Also, it was regulated by the regulation constant values, obtained from mean value of signal. The PI controller adjusted the high deviations of attitude and removed the error diverges, and it aligned the predicted error with the error signal. Finally, the regulation constant values removed the bias of the estimated error. The result of RMSE for each attitude channel is mentioned in Table 3.

### 4.2. Dynamic Test and Results

**Figure 7:**
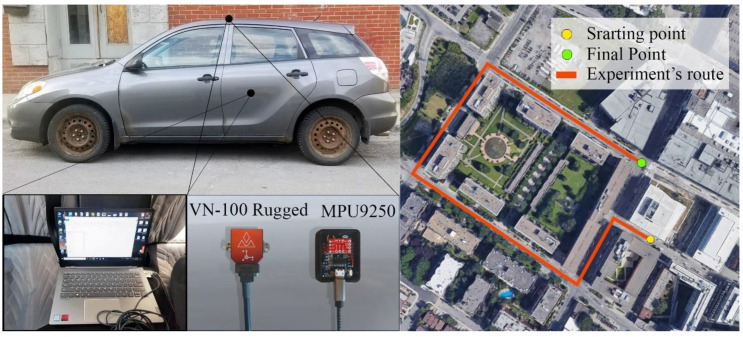
Dynamic experiment’s equipment and the experiment’s path.

A dynamic test was designed to validate the performance of the proposed method in a real dynamic situation. A regular ground vehicle was selected to perform the experiment in the marked path. The experiment was performed in a region of Montreal. This time, the orientation reference was measured by a VN-100 Rugged IMU and AHRS board with the specifications pointed in Table 4. The MPU-9250 and VN-100 Rugged AHRS reference were mounted on top of the car, and they were connected to a laptop to record the true and measured data with two USB cables. The VN-100 Rugged board was connected to the VectorNav software as a control center Graphic User Interface (GUI). Figure 7 demonstrates the location and experiment’s route with starting and final points and shows the equipment used in this experiment.

**Figure 8:**
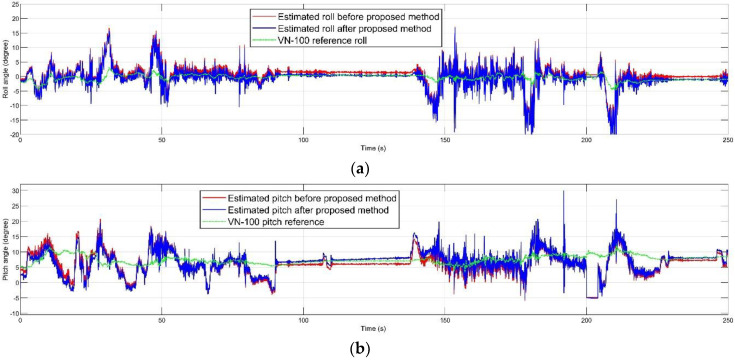

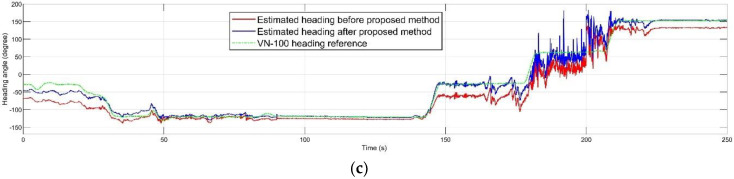
Estimated orientation before and after performing the propsed method compared to the true reference orientation: (**a**) roll; (**b**) pitch; (**c**) heading.

The mentioned dynamic accuracy experiment was performed in 250 s. The inertial and magnetic data of the MPU-9250 and the AHRS reference data of VN-100 board were recorded during the experimental path. The experiment’s route was selected according to smooth inclination. Figure 8 demonstrates the attitude estimation before and after the proposed method compared to the true reference.

**Figure 9:**
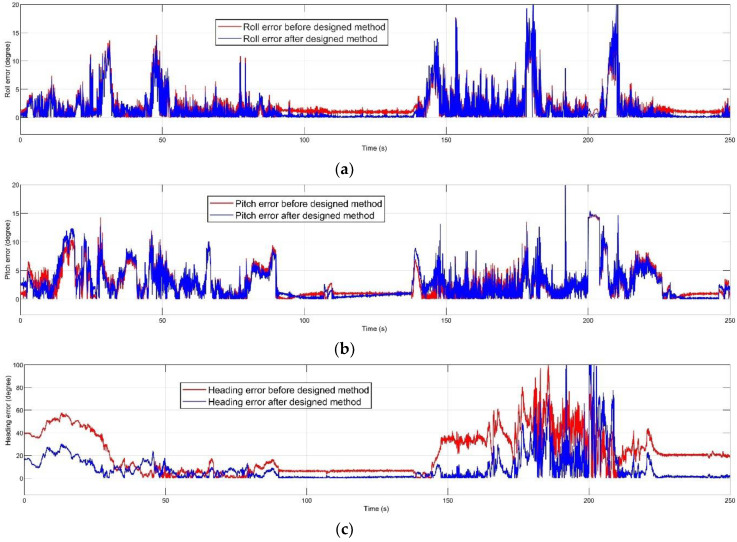
Absolute attitude error before and after performing the method for each orientation channel: (**a**) roll; (**b**) pitch; (**c**) heading.

**Figure 10:**
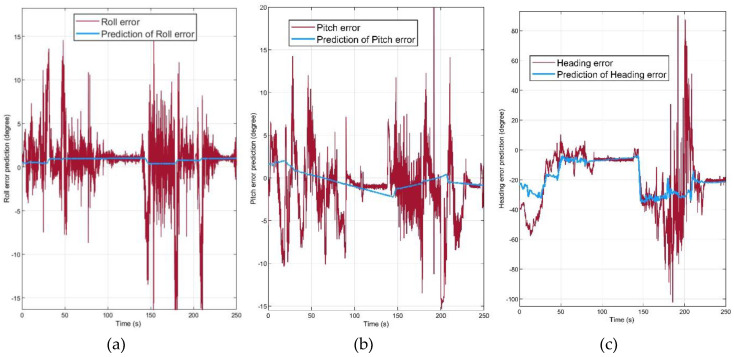
(**a**) Roll error and roll error prediction signal; (**b**) pitch error and pitch error prediction signal; (**c**) heading error and heading error prediction signal.

Figure 9 illustrates the absolute orientation error before and after the error prediction method. The proposed algorithm had the most significant effect on the heading error, and the error in roll and pitch was decreased. Figure 10 demonstrates that the attitude error prediction filter proposed in this paper could estimate the orientation error. The prediction signal for each channel was obtained after the PI controller and regulation.

These prediction signals were obtained with defining the parameters of PI controller and the regulator. The proportional gain, integral gain, and regulation constant values are defined in the Table 5 for the described dynamic experiment. By determining these parameters as Table 5, the most optimum error prediction signals were obtained.

The RMSE was calculated for each orientation channel, as described in Table 6. The static test was more challengeable for the roll and the pitch channels because of more movement along the x and y axes in the designed static experiment. However, due to the dynamic test with ground vehicle, the heading was more affected in this experiment. After performing the proposed AHRS error prediction method, the RMSE result for the roll and the pitch channels were decreased about 80% and 43%, respectively, and the heading’s precision improved by approximately 66%.

The Figure 6 and Figure 9 show the results of error compensation. However, it can be seen that in some phases of the trajectories, the attitude errors were not corrected. To analyze this, we need to consider the essence of the PI controller. The PI controller created some oscillations on the output of the system due to the integration part. Therefore, the error prediction system demonstrates the better performance in the overall investigation than in short-term phases. In other words, the PI controller tuned the predicted error signal to have the overall robust and stable attitude, which may have led to the lack of change in some parts.

## 5. Conclusions

The designed error prediction and compensation filter were implemented and tested with a low-cost IMU module. Two experiments were designed to validate the performance of designed method, one for static and one for dynamic validation. In the static test, the performance was compared to an iPhone smartphone orientation reference, the method demonstrated between 65% and 80% accuracy improvement in all orientation channels. For the dynamic test, the precise AHRS determiner board was selected as a true reference and the experiment was performed with ground vehicle, which showed about 60–80% error deduction in roll and heading, and about 43% accuracy improvement in pitch channel. Consequently, the proposed method demonstrated the significant improvement in the roll and the heading and moderate progress in the pitch estimation.

Although the presented method showed progress in error reduction of all channels in both static and dynamic tests, the error prediction can be modified to use other inertial sensors apart from the gyroscope. Also, because the method extremely depends on parameters of PI controller and regulator, finding a reliable mathematical solution for calculating them can be investigated for more accurate results. Determining the overshooting, overall stability, and other parameters for transfer function of the PI controller can play an active role in designing the prediction filter. Furthermore, the possibility of real-time calibration by estimating the parameters of error prediction filter warrants future investigations.

## Figures and Tables

**Table 1:** Comparison of error between iPhone 11 and Samsung S10’s AHRS algorithms.

Angle	Samsung S10	iPhone 11
Roll’s RMSE	0.3801°	0.2312°
Pitch’s RMSE	0.4117°	0.3993°
Yaw’s RMSE	1.2313°	0.7388°

**Table 2:** Parameters of PI controller and regulator for each static orientation experiment.

Experiment	Proportional Gain ($K_{P}$)	Integral Gain ($K_{I}$)	Regulation Constant (ε)
Roll experiment	$K_{P\_1}=-19$	$K_{I\_1}=0.002$	$\varepsilon_{1}=-2$
Pitch experiment	$K_{P\_2}=-75$	$K_{I\_2}=0.5$	$\varepsilon_{2}=2$
Heading experiment	$K_{P\_3}=-2250$	$K_{I\_3}=0.002$	$\varepsilon_{3}=24$

**Table 3:** RMSE of roll, pitch, and heading before and after the proposed filter in static test.

Channel	Before Performing the Filter	After Performing the Filter	Static Accuracy Improvement
Roll’ RMSE	1.7603°	0.4298°	75.58%
Pitch’s RMSE	3.7735°	0.7268°	80.74%
Heading’s RMSE	1.1232°	0.3720°	66.88%

**Table 4:** Specification of the VN-100 Rugged AHRS board.

Dynamic Accuracy (Heading)	Dynamic Accuracy (Roll and Pitch)	Attitude Output Rate	Operation Temperature	Baud Rate
2.0° RMS	1.0° RMS	400 Hz	–40°C to 85 °C	Up to 921,600

**Table 5:** Parameters of PI controller and regulator for each orientation experiment in the dynamic test.

Channel	Proportional Gain ($K_{P}$)	Integral Gain ($K_{I}$)	Regulation Constant (ε)
Roll	$K_{P\_1}=-19$	$K_{I\_1}=0.002$	$\varepsilon_{1}=-0.7$
Pitch	$K_{P\_2}=50$	$K_{I\_2}=0.05$	$\varepsilon_{2}=-1.1$
Heading	$K_{P\_3}=-4000$	$K_{I\_3}=-0.001$	$\varepsilon_{3}=-17$

**Table 6:** Root mean square error (RMSE) of roll, pitch, and heading before and after proposed filter in dynamic test.

Experiment	Before Performing the Filter	After Performing the Filter	Static Accuracy Improvement
Roll’s EMSE	0.9220°	0.1880°	79.61%
Pitch’s RMSE	0.8611°	0.4889°	43.22%
Heading’s RMSE	22.6029°	7.6072°	66.34%
